# Mediating Effect of Job Burnout on the Relationship Between Organisational Support and Quiet Quitting in Nurses

**DOI:** 10.1111/jan.16599

**Published:** 2024-11-05

**Authors:** İbrahim Gün, Habip Balsak, Fatma Ayhan

**Affiliations:** ^1^ Department of Health Management, Faculty of Health Sciences Batman University Batman Turkey; ^2^ Midwifery Department, Faculty of Health Sciences Batman University Batman Turkey; ^3^ Division of Psychiatric Nursing, Department of Nursing, Faculty of Health Science Batman University Batman Turkey

**Keywords:** job burnout, nursing, organisational support, quiet quitting

## Abstract

**Aim:**

The main purpose of this study was to investigate the potential mediating role of job burnout in the relationship between organisational support and quiet quitting among nurses. Additionally, this study aimed to determine the associations between organisational support, job burnout and quiet quitting.

**Design:**

This study was a descriptive, cross‐sectional study.

**Methods:**

This descriptive and cross‐sectional study included a total of 383 nurses. The convenience sampling method was used, and the study was conducted in Türkiye. Self‐reported measures, which included organisational support, job burnout and the quiet quitting scale, were completed by using an online version of the scale.

**Results:**

Statistically significant associations were found between organisational support, job burnout and quiet quitting. Organisational support had a significant negative effect on quiet quitting. Additionally, job burnout had a positive effect on quiet quitting behaviour. Moreover, job burnout partially mediated the relationship between organisational support and quiet quitting.

**Conclusion:**

The findings highlight the importance of considering job burnout as a critical factor in mitigating the positive effect of organisational support on quiet quitting. Prioritising the job burnout of healthcare workers plays a significant role in reducing quiet quitting behaviour through organisational support.

**Impact:**

This study focused on how organisational support and burnout interact with quiet quitting, which is a current issue among nurses, and explained the mediating role of job burnout in the relationship between organisational support and quiet quitting. The main findings of this research provide evidence that organisational support influences quiet quitting behaviour. Similarly, job burnout affects quiet quitting behaviour. Moreover, job burnout plays a mediating role in the relationship between organisational support and quiet quitting behaviour. It has been proven that job burnout is a significant barrier to the impact of organisational support in reducing quiet quitting behaviour. This research will have an impact on the management and strategic planning of healthcare organisations.

**Reporting Method:**

STROBE reporting method has been followed.

**Patient or Public Contribution:**

No patient or public contribution.

## 1 Introduction

Quiet quitting, which has emerged as a much‐discussed phenomenon in the workplace since the COVID‐19 pandemic, has gained prominence in recent discussions. This concept, which first emerged on social media, is relatively new to the literature. Quiet quitting is defined as an employee's partial withdrawal from his/her responsibilities in the workplace or not going beyond his/her job descriptions without clearly expressing his/her turnover intention (Scheyett 2023). While there are various definitions in the literature, a quiet quitter is generally described as an employee who chooses to continue his or her job despite having turnover intention, often due to conjunctural circumstances (Hiltunen 2023). Therefore, the person fulfils his/her duties at the workplace at a minimum level. In 2022, when extensive studies were conducted, more than half of the employees in the USA exhibited quiet quitting behaviour (Hamouche, Koritos, and Papastathopoulos 2023). Some statistical data even report that this rate affects 85% of the working population (Liu‐Lastres, Karatepe, and Okumus 2024). The prevalence of this newly defined phenomenon in the business world has increased interest in investigating the causes and consequences of quiet quitting. Researches indicate that burnout as an individual cause (Galanis, Moisoglou, Katsiroumpa, et al. 2024; Moon, O'Brien, and Mann 2023) and managerial organisational support (Gabelaia and Bagociunaite 2023) are important reasons for quiet quitting behaviour. Studies conducted in various sectors have identified a lack of appreciation from management and the absence of adequate career opportunities as significant factors contributing to the emergence of quiet quitting behaviour (Pevec 2023). Eventually, the most common cause of quiet quitting behaviour, which has various organisational and individual determinants, can be summarised as a lack of employee autonomy and career opportunities, a perceived decline in employee value and a decrease in organisational trust (Mahand and Caldwell 2023). All of these findings are significant in highlighting the relationship between organisational support and burnout as underlying causes of quiet quitting.

Quiet quitting in the healthcare sector was reported to impact patient care and employee well‐being and emerge as a result of numerous negative factors (Galanis et al. 2023; Pevec 2023). High levels of stress, work overload and burnout among healthcare workers are identified as significant contributors to the prevalence of quiet quitting in the healthcare sector (Kang, Kim, and Cho 2023; Pevec 2023). However, hospitals are organisations with very complex structures where different services are carried out together. These unique characteristics can lead to management challenges that negatively impact employees. The inability to carry out effective leadership in the working environment and the lack of managerial support encourage health workers to seek alternative job opportunities (Pevec 2023). The absence of adequate career opportunities in the workplace prompts healthcare professionals to seek more fulfilling options elsewhere for their professional development (Salehi et al. 2020). In addition to the aforementioned factors, the disruption of work–life balance due to long and demanding working conditions is a significant risk factor for the emergence of quiet quitting among healthcare workers (Pevec 2023).

Nurses, constituting more than half of the healthcare workforce, constitute the cornerstone of patient care and treatment. Healthcare professionals also endure the longest working hours (Watts, Robertson, and Winter 2013). The prevalence of quiet quitting among nurses poses a substantial threat to the healthcare system, considering their critical role in patient care and the long working hours they typically endure (Galanis et al. 2023). This situation is likely to result in significant adverse effects on the delivery of healthcare services, particularly on the quality of patient care. Furthermore, the consideration of quiet quitting among nurses, who play a crucial role within healthcare teams, undermines team cohesion and trust and may disrupt a positive work environment (Kang, Kim, and Cho 2023). The findings presented underscore the importance of establishing clear cause–and–effect relationships to effectively understand and prevent the quiet quitting phenomenon among nurses.

The behaviour of quiet quitting has been identified at a rate of 85% across different sectors (Liu‐Lastres, Karatepe, and Okumus 2024), while this rate is reported as 60.9% among nurses specifically (Galanis et al. 2023). Detecting quiet quitting behaviour is difficult because employees tend to conceal it. Therefore, it is believed that the prevalence of quiet quitting behaviour exceeds the currently detected rates. Additionally, it has been reported that quiet quitting is expected to become more widespread among nurses in the future (Galanis, Moisoglou, Katsiroumpa, et al. 2024). The growing prevalence of quiet quitting among nurses poses a significant threat to the nursing workforce and, consequently, the healthcare system. Therefore, ensuring the well‐being of nurses and increasing their work engagement is essential in terms of providing optimal patient care and preventing other negativities related to quiet quitting (Kang, Kim, and Cho 2023). Furthermore, identifying the primary factors causing quiet quitting and implementing targeted measures to retain the nursing workforce is crucial for healthcare organisations.

In addition to the strong correlation between organisational support and burnout, different studies have also demonstrated the mediating role of organisational support in quiet quitting (Moon, O'Brien, and Mann 2023; Tsemach and Barth 2023). There is a moderate negative correlation between organisational support and nurses' turnover intention. In other words, nurses who receive greater organisational support are less likely to consider leaving their jobs compared to those who receive less support (Galanis, Moisoglou, Papathanasiou, et al. 2024). Although, numerous studies in the literature have examined the relationship between organisational support and turnover intention, to our knowledge, no studies have investigated the relationship between organisational support and quiet quitting among nurses. A study conducted on Generation Z in China revealed that perceived organisational support reduces quiet quitting behaviour and that occupational burnout played a mediating role in this relationship. As job burnout increases quiet quitting intention of Generation Z increases. Furthermore, job burnout mitigates the positive effect of organisational support in reducing quiet quitting intention (Xueyun et al. 2023). However, to the best of our knowledge, no study has investigated how organisational support and job burnout affect and how job burnout mediates the relationship between organisational support with quiet quitting intention of nurses. On the basis of the literature mentioned above, this study aims to explore the mediating role of job burnout in the relationship between organisational support with quiet quitting among nurses. On the basis of these aims, the following hypotheses are formulated: (i) Organisational support would have direct effect on job burnout and quiet quitting. (ii) Job burnout would have direct effect on quiet quitting. (iii) Job burnout would mediate the association of organisational support with quiet quitting.

## 2 Method

This study was designed as a descriptive, cross‐sectional study, and the data were collected between December 2023 and April 2024.

### 2.1 Participants

The participants were selected using convenience sampling method. A total of 383 nurses in Türkiye participated in the study. The median age of the participants was 30 years. The nurses consisted of 71.02% females and 28.98% males, with the majority reporting being married (61.36%), followed by being single (38.64%). The majority of the participants had a bachelor's degree (45.69%), followed by an associate degree (28.98%), a master's degree (13.32%) and a high school degree (12.01%). The working units of nurses varied, with the majority working in the emergency room (19.58%), followed by internal clinics (16.45%) and surgical clinics (15.40%). Among the nurses, 63.97% worked 40 h per week. In terms of years of professional experience, 35.77% of the nurses had 1–5 years of experience, 29.77% had 6–10 years and 34.46% had 11 or more years of experience. The majority of the participants (54.31%) stated that their income was less than their expenses. A full description of the participants is presented in Table 1.

**TABLE 1:** Demographic characteristics of participants.

Variables	Frequency	Percent
Age (median age: 30)		
20–27 years	124	32.38
28–32	116	30.29
33 and more	143	37.34
Gender		
Female	272	71.02
Male	111	28.98
Marital status		
Single	148	38.64
Married	235	61.36
Education		
High school	46	12.01
Associate degree	111	28.98
Bachelor's degree	175	45.69
Master degree	51	13.32
Unit		
Emergency room	75	19.58
Surgical units	37	9.66
Intensive care unit	45	11.75
Internal clinics	63	16.45
Surgical clinics	59	15.40
Administration units	44	11.49
Others^a^	60	15.67
Type of shift		
Day shift	237	61.88
Duty shift	146	38.12
Weekly working hours		
40	245	63.97
41–45	71	18.54
46 and more	67	17.49
Years of professional experience (years)		
1–5	137	35.77
6–10	114	29.77
11 and more	132	34.46
Income status		
Income is less than expenses	208	54.31
Income equals expenses	148	38.64
Income exceeds expenses	27	7.05
Total	383	100.00

^a^ Others: Family Health Center, Community Health Center, Community Mental Health Center, Cancer Early Diagnosis, Screening and Education Center.

### 2.2 Measures

In the present study, a package of questionnaire form, including demographic questions, organisational support, job burnout and quiet quitting scale, was used.

#### 2.2.1 Sociodemographic Variables

The sociodemographic variables used in the study were as follows: age, sex, marital status, education level, unit, type of shift, weekly working hours, years of professional experience and income status.

#### 2.2.2 Organisational Support Scale

The organisational support scale was developed by Eisenberger et al. (1986). Short form of the scale adopted into the Turkish healthcare sector by Türe and Yıldırım (2018). The participants rated nine self‐reported items on a 5‐point Likert scale ranging from 1 (Strongly disagree) to 5 (Strongly agree). Higher sore indicates that higher organisational support by management. The Cronbach's alpha of the scale was 0.88. In the present study, it was 0.83. A sample item is ‘The organization I work for is proud of my achievements’.

#### 2.2.3 Job Burnout Scale

To determine levels of job burnout, we utilised a scale that assesses self‐reported physical, emotional and mental exhaustion through 10 items in the last 2 weeks. A sample item of the scale is ‘Tired’. Participants rated these items on a 7‐point Likert scale ranging from 1 (never) to 7 (always). The burnout scale was developed by Pines and Aronson (1988) and consists of 21 items. Pines (2005) adapted its 10‐item shorter form as an easy‐to‐use instrument composed of fewer items to meet the needs of researchers and practitioners. The Turkish validation of the scale was conducted by Çapri (2013). The scale demonstrated a high level of reliability, with a Cronbach's alpha exceeding 0.85. In our study, the Cronbach's alpha for the scale was 0.92, indicating acceptable internal consistency. Higher scores on the scale are indicative of greater job‐related burnout.

#### 2.2.4 Quiet Quitting (QQ) Scale

The quiet quitting scale was developed by Anand, Doll, and Ray (2023). The scale has eight self‐reported items, ranging from 1 (Strongly disagree) to 5 (Strongly agree). A sample item of the scale was ‘I often arrive late and leave early from work’. The Cronbach's alpha of the scale was 0.829. Higher score obtained from the scale indicates high level of quiet quitting. In our research, the quiet quitting scale developed by Anand, Doll, and Ray (2023) was introduced to the Turkish context for the first time, ensuring linguistic validity through rigorous procedures, including exploratory factor analysis and confirmatory factor analysis. The study reported a high level of internal consistency, with Cronbach's alpha reaching 0.82. Confirmatory factor analysis further supported the reliability of the measurement model, as evidenced by favourable indices: CIMIN/DF = 2.391, CFI = 0.976, TLI = 0.963, RMSEA = 0.060 and SRMR = 0.036. Standardised factor loadings, ranging from 0.55 (item 2) to 0.87 (item 5), underscore the validity and reliability of the scale. In alignment with Hu and Bentler's (1999) criteria, the results indicated a strong model fit, affirming the scale's reliability in measuring the concept of quiet quitting in the Turkish context.

### 2.3 Procedure

In the beginning, the requisite permissions for the utilisation of the scales in this study were obtained from the researchers through email. Then, ethical committee approval was received from the Batman University Ethics Committee (E‐135537/2023‐06‐03). Informed consent was obtained from the participants online. On the first page of the website, participants were provided with information about the study. Those who wished to participate were asked to confirm the statement, ‘I agree to participate in the research’, and only those who gave consent were able to take part in the study. Additionally, the data were collected in accordance with the Helsinki Declaration. This study used an online version of the scales to collect the data. A package of questionnaires included information questions and scales. The scales take approximately 10 min to complete. The convenience sampling method was used in the present study. One of the inclusion criteria for the study was having worked as a nurse for at least 1 year. The data used in the study were collected between December 2023 and April 2024. During data collection, personal social media accounts (Facebook, Instagram, WhatsApp and Twitter) were utilised. Information related to the subject and purpose of the study was shared through social media accounts, and an invitation link was shared. Informed consent was obtained from the participants who agreed to participate in the study, and they were assured of the confidentiality and anonymity of their responses.

### 2.4 Data Analysis

A total of 383 nurses participated in the present study. The sufficiency of the sample size was assessed on the basis of the recommendation of Fritz and MacKinnon (2007). The sample size was greater than the recommended range of 115–285. Before proceeding with the primary analysis, descriptive statistics, such as mean and standard deviations, were calculated. These analyses aimed to assess the characteristics of the scales and verify the normality assumption for the study variables. The assumption of normality of the data was examined by analysing the skewness and kurtosis scores (Tabachnick, Fidell, and Ullman 2013). On the basis of the normality test with skewness and kurtosis scores, the data were distributed normally. Pearson correlation analysis was performed to explore the relationships between organisational support, quiet quitting and job burnout. Afterwards, the mediation model was performed through utilising PROCESS v4.0 for Windows (Model 4). The confidence interval was assumed to be 95% at a significance level of *p* < 0.05, and the number of bootstrap samples for percentile bootstrap confidence intervals was 5000 (Preacher and Hayes 2008). Model 4 was used to figure out the relationship between independent variable and dependent variable through mediation analysis. In the present study, a mediation model was tested to explore the mediating role of job burnout in the relationship between organisational support and quiet quitting. The outcomes of the mediation analysis were assessed through the examination of squared‐multiple correlations (*R* ^2^) and standardised regression estimates (*β*). These metrics provided a comprehensive evaluation of the explanatory power of the model and the strength of the relationships between variables (Yıldırım et al. 2023). All the statistical analyses were performed by using SPSS v25.

## 3 Results

### 3.1 Preliminary Findings

Before the mediation model was assessed, preliminary analyses were conducted, including descriptive statistics, reliability measures, an examination of normal distribution assumptions and the calculation of correlation coefficients. The assumption of a normal distribution was tested on the basis of the rule of thumb of ±2 suggested by George and Mallery (2010) and Tabachnick, Fidell, and Ullman (2013). According to the findings, the data were normally distributed. The Cronbach's alpha coefficients of the organisational support, job burnout and quiet quitting scales were 0.83, 0.92 and 0.82 respectively. All the preliminary findings are presented in Table 2. The results of the correlation analysis revealed that organisational support was negatively and significantly correlated with job burnout (*r* = −0.519, *p* < 0.01) and quiet quitting (*r* = −0.527, *p* < 0.01). Additionally, job burnout demonstrated a significant and positive correlation quiet quitting (*r* = 0.628, *p* < 0.01). The correlation analysis results are presented in Table 2.

**TABLE 2:** Descriptive statistics and correlations between the study variables.

Variable	Mean	SD	Skewness	Kurtosis	*α* **	1	2	3
1. Organisational support	2.81	0.81	0.12	−0.36	0.83	—	−0.519*	−0.527*
2. Job burnout	3.79	1.73	0.29	−0.23	0.92		—	0.628*
3. Quiet quitting	2.62	0.93	−0.14	−0.69	0.82			—

Abbreviation: SD, standard deviation.

* *p* < 0.05. All correlations are significant at the 0.001 level (two‐tailed).

** *α* = Cronbah's alpha reliability coefficient.

### 3.2 Mediation Analyses

**FIGURE 1:**
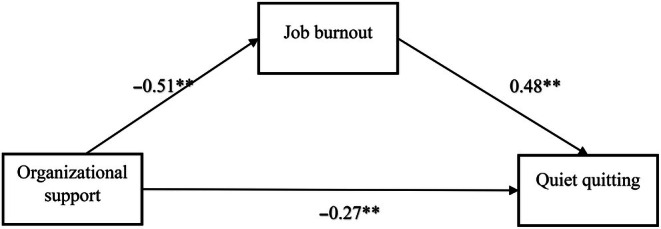
Conceptual model of the hypothesised associations among the study variables. **Standardised coefficient values, *p* < 0.05.

Mediation analyses were carried out to investigate the possible mediating role of job burnout in the relationship between organisational support and quiet quitting. The results of the mediation analysis revealed that organisational support displayed a significant predictive effect on nurses' job burnout (*β* = −0.518, *p* < 0.001) and quiet quitting (*β* = −0.274, *p* < 0.001). Additionally, job burnout displayed a significant predictive effect on quiet quitting (*β* = 0.485, *p* < 0.001) (see Figure 1). Organisational support explained 27% of the variance of job burnout. Together, organisational support and job burnout explained 45% of the variance of the quiet quitting.

The direct effect of organisational support on quiet quitting was significant (*β* = −0.314, *p* < 0.001). Notably, the mediation analysis demonstrated a significant indirect effect of organisational support on quiet quitting through job burnout. The indirect effect of organisational support on quiet quitting (*β* = −0.288, 95% CI [−0.312, −0195]) through job burnout was significant since the 95% confidence interval did not contain zero.

When the mediating effect of job burnout on the effect of organisational support on quiet quitting was examined, the level of the indirect effect was negative and significant because the BootLLCI and BootULCI values did not contain 0. Standardised and unstandardised predictive effects showing the associations between the analysed variables are presented in Tables 3 and 4. The results indicated that job burnout partially mediated the relationship between organisational support and quiet quitting. Job burnout plays a mediating role in the effect of organisational support on quiet quitting, and as the level of burnout among nurses increases, the positive effect of organisational support on preventing quiet quitting decreases. Further indirect effect details can be found in Table 4.

**TABLE 3:** Unstandardised coefficients for the mediation model.

Antecedent	Consequent							
	*M* (Job burnout)				*Y* (Quiet quitting)			
	Coeff.	SE	*t*	*p*	Coeff.	SE	*t*	*p*
*X* (organisational support)	−1.109	0.093	−11.844	< 0.001	−0.314	0.050	−6.171	< 0.001
*M* (job burnout)					0.259	0.023	10.906	< 0.001
Constant					2.523	0.207	12.136	< 0.001
	*R* ^2^ = 0.269				*R* ^2^ = 0.449			
	*F* = 140.287; *p* < 0.01				*F* = 155.200; *p* < 0.01			

*Note:* Number of bootstrap samples for percentile bootstrap confidence intervals: 5000.

Abbreviations: Coeff, unstandardised coefficient; *M*, mediator variable; SE, standard error; *X*, independent variable; *Y*, outcomes variable.

**TABLE 4:** Direct and indirect effects of organisational support on quiet quitting.

	Unstandardised coefficient	BootLLCI	BootULCI	Standardised coefficient
Direct effect	−0.314*	−0.414	−0.214	−0.274
Indirect effect through job burnout (*M*)	−0.288*	−0.362	−0.220	−0.251
Total effect	−0.602*	−0.700	−0.504	−0.526

* *p* < 0.01.

## 4 Discussion

Research on quiet quitting is still in its early stages and the literature on quiet quitting is quite limited. The literature highlights that the rate of quiet quitting among nurses is quite high, at approximately 60.9%, and this rate is expected to increase even further in the future (Galanis, Moisoglou, Malliarou, et al. 2024). In studies conducted among nurses, the effects of turnover intention (Galanis, Moisoglou, Malliarou, et al. 2024), burnout and job satisfaction (Galanis et al. 2023) and the influence of bullying and negative coping strategies on quiet quitting were assessed (Galanis, Moisoglou, Katsiroumpa, et al. 2024). However, to date, no research has explored the impact of perceived organisational support on quiet quitting among nurses, nor has the mediating role of burnout been examined. Therefore, our research findings provide foundational knowledge for the literature on quiet quitting. Our study examined the mediating effect of job burnout on the relationship between organisational support and quiet quitting among nurses.

Our research findings confirmed our initial hypothesis that organisational support would have a direct effect on nurses' job burnout and quiet quitting. Additionally, job burnout was found to be a significant predictor of quiet quitting. There is plenty of evidence in the literature suggesting that high organisational support positively impacts job satisfaction (Liu et al. 2018; Page and Graves 2021; Patrick and Laschinger 2006; Tang et al. 2023), turnover intention (Abou Hashish 2017; Kwak et al. 2010; Li et al. 2020; Lowe et al. 2020), organisational commitment (Gupta, Agarwal, and Khatri 2016) and burnout (Kwak et al. 2010; Zheng and Wu 2018) of nurses. A systematic review investigating the factors influencing quiet quitting identified several key contributors. These include employees feeling undervalued, a lack of organisational investment in career growth opportunities, disengagement from their work, being excluded from important decision‐making processes, limited autonomy in their roles and a diminishing sense of trust in the organisation. All of these factors significantly contribute to the rise in quiet quitting behaviours among employees (Pevec 2023). These factors are indicative of some aspects of organisational support, thus supporting our findings regarding the impact of organisational support on quiet quitting. Nurses play an exceptionally crucial role within the medical system and constitute approximately 60% of the healthcare workforce (Soto‐Rubio, Giménez‐Espert, and Prado‐Gascó 2020). Moreover, nurses have the longest working hours among all medical and healthcare professionals, making them more vulnerable to job burnout (Sousa et al. 2020; Watts, Robertson, and Winter 2013). A high level of organisational support through aspects such as justice, managerial support, organisational rewards and suitable working conditions helps enhance and increase nurses' job satisfaction and positive emotions. Nurses with a high sense of organisational support feel satisfied with their organisations, which, in turn, translates into a sense of loyalty to the organisation (Ahmad et al. 2022). On the basis of these findings, organisations should strengthen communication, provide training and development opportunities, establish a feedback culture, promote teamwork and provide rewards as strategies to increase organisational support and reduce nurses' levels of burnout and quiet quitting.

The research findings confirm our second hypothesis that burnout has a direct effect on quiet quitting. In the limited number of studies conducted with nurses, burnout has been associated with increased absenteeism, turnover intention and quiet quitting (Galanis et al. 2023; Sasso et al. 2019). While quiet quitting may be chosen as a response to challenging work conditions, it ultimately appears to be a temporary solution, and nurses who opt for quiet quitting are still more likely to intend to leave their jobs (Galanis, Moisoglou, Malliarou, et al. 2024). Additionally, Boy and Sürmeli (2023) stated that burnout resulting in quiet quitting. Since quiet quitting is related to the work–life balance of healthcare workers, understanding it could aid in addressing psychological issues such as depression, anxiety, posttraumatic stress disorder, burnout and suicide among healthcare workers.

Our third hypothesis was that burnout would mediate the relationship between organisational support and quiet quitting, and our findings supported this hypothesis. Organisational support has a partial mediating effect on the impact of quiet quitting, and as the level of burnout increases in nurses, the positive effect of organisational support in preventing quiet quitting decreases. In parallel with our findings, research conducted among Z generation in China found that organisational support affects quiet quitting and that job burnout plays a mediating role in the relationship between organisational support and quiet quitting (Xueyun et al. 2023). The literature has demonstrated that organisational commitment among healthcare professionals reduces burnout (Gorgulu and Akilli 2017; Jun et al. 2021), and high job satisfaction is associated with lower turnover intention (Al Sabei et al. 2020). Another recent study reported that experiencing burnout increases the rate of quiet quitting among nurses and that job satisfaction plays a mediating role between burnout and quiet quitting (Galanis et al. 2023). Individuals working in demanding and stressful professions such as nursing often experience emotional exhaustion (Galanis, Moisoglou, Katsiroumpa, et al. 2024; Kwak et al. 2010; Zheng and Wu 2018). This study and other studies on this topic demonstrate that organisational support is an effective tool for reducing burnout level. Another key point is that organisational support plays a critical role in preventing quiet quitting. As organisational support increases, employees feel happier and more satisfied at work, which can help prevent quiet quitting. The results obtained from the present study demonstrated that job burnout diminishes the positive impact of organisational support on quiet quitting. To reduce quiet quitting behaviour, organisational support should be provided to health workers, and their job burnout should not be ignored.

## 5 Implication and Limitations

Quiet quitting is a new phenomenon that has attracted attention worldwide in recent years, and studies are still limited. To the best of our knowledge, this is the first study conducted among nurses. The unique characteristics of our research approach and findings hold significant implications and shed light on understanding and managing quiet quitting among nurses in Türkiye. The pervasiveness of quiet quitting among nurses, particularly those bearing the brunt of hospital workloads, poses a significant challenge to healthcare systems worldwide. Addressing this issue effectively has the potential to yield a multitude of positive outcomes, including elevated nurse motivation and engagement, reduced healthcare costs, improved patient satisfaction and enhanced reputation and recruitment. Additionally, our findings could assist policymakers or decision‐makers in making more effective decisions on the matter. However, our research has several limitations. First, since our research data were collected on online platforms, nurses who do not use social media accounts may not have been reached. However, the use of a convenience sampling method resulted in a greater inclusion of easily accessible nurses in the study. Within the scope of the study, the greater interest shown by emergency department nurses partially affected the diversity of the sample. Second, the study was conducted cross‐sectionally, which means that a clear picture of the long‐term status of quiet quitting among nurses cannot be determined. Another limitation is that the data were collected on the basis of self‐reports from nurses, without individual interviews with nurses or observations of their work conditions in the workplace. Finally, while our research sheds light on the potential influence of organisational support and burnout on quiet quitting among nurses, it is likely that other factors also play a significant role.

## 6 Conclusions

The findings of our research have significant implications for organisational practices and policymaking in the healthcare sector. Our findings highlight the significant impact of organisational support on burnout and quiet quitting among nurses, indicating the need for healthcare organisations to consider these factors in their management and strategic planning. First, leaders can develop various strategies to increase nurses' access to organisational support in the workplace. Second, adopting preventive and interventional approaches is crucial for reducing occupational burnout. Initiatives such as stress management programs, workload balancing and support groups to reduce emotional strain can help decrease burnout levels among nurses, potentially reducing their inclination towards quiet quitting. The well‐being and satisfaction of nurses in the workplace are directly linked to the quality of healthcare and patient care. Therefore, implementing effective measures to address the needs of their employees is crucial for managers and policymakers. Quiet quitting has become a hot topic in the world of work in recent times. To better understand this phenomenon and achieve better outcomes for both employers and employees, longitudinal and observational modelling research would be highly beneficial. In future studies, the moderating effect of organisational support on the relationship between job burnout and quiet quitting could be examined, contributing to the enrichment of the literature on the concept of quiet quitting. Additionally, since many factors may mediate or covariate the relationship between organisational support and quiet quitting intention, further research is needed to better understand the conceptual framework by examining variables such as job satisfaction, well‐being, work stress, work conditions, job security and organisational commitment. Furthermore, the use of stratified sampling methods is recommended in populations where stratification is possible.

## Ethics Statement

The ethical committee approval was received from Batman University Ethics Committee (E‐135537/2023‐06‐03).

## Consent

Informed consent was obtained from the participants who agreed to participate in the study, and they were assured of confidentiality and anonymity of responses.

## Conflicts of Interest

The authors declare no conflicts of interest.

## Peer Review

The peer review history for this article is available at https://www.webofscience.com/api/gateway/wos/peer‐review/10.1111/jan.16599.

## Permission to Reproduce Material From Other Sources

In the beginning, requisite permissions for the utilisation of the scales in this study were obtained from the researchers through email.

## Data Availability

Research data can be requested by contacting the researchers.

## References

[jan16599-bib-0001] Abou Hashish, E. A. 2017. “Relationship Between Ethical Work Climate and Nurses' Perception of Organizational Support, Commitment, Job Satisfaction and Turnover Intent.” Nursing Ethics 24, no. 2: 151–166. 10.1177/0969733015594667. 26260440

[jan16599-bib-0002] Ahmad, M. S. , M. Barattucci , T. Ramayah , T. Ramaci , and N. Khalid . 2022. “Organizational Support and Perceived Environment Impact on Quality of Care and Job Satisfaction: A Study With Pakistani Nurses.” International Journal of Workplace Health Management 15, no. 6: 677–693. 10.1108/IJWHM-09-2021-0179.

[jan16599-bib-0003] Al Sabei, S. D. , L. J. Labrague , A. Miner Ross , et al. 2020. “Nursing Work Environment, Turnover Intention, Job Burnout, and Quality of Care: The Moderating Role of Job Satisfaction.” Journal of Nursing Scholarship 52, no. 1: 95–104. 10.1111/jnu.12528. 31692251

[jan16599-bib-0004] Anand, A. , J. Doll , and P. Ray . 2023. “Drowning in Silence: A Scale Development and Validation of Quiet Quitting and Quiet Firing.” International Journal of Organizational Analysis, Ahead‐of‐Print 32: 721–743. 10.1108/IJOA-01-2023-3600.

[jan16599-bib-0005] Boy, Y. , and M. Sürmeli . 2023. “Quiet Quitting: A Significant Risk for Global Healthcare.” Journal of Global Health 13: 03014. 10.7189/jogh.13.03014. 36995298 PMC10062397

[jan16599-bib-0006] Çapri, B. 2013. “Tükenmişlik ölçeği‐kısa formu ile eş tükenmişlik ölçeği‐kısa formu'nun türkçe uyarlaması ve psikoanalitik‐varoluşçu bakış açısından mesleki ve eş tükenmişlik ilişkisi.” Kuram ve Uygulamada Eğitim Bilimleri 13, no. 3: 1393–1418.

[jan16599-bib-0007] Eisenberger, R. , R. Huntington , S. Hutchison , and D. Sowa . 1986. “Perceived Organizational Support.” Journal of Applied Psychology 71, no. 3: 500–507. 10.1037/0021-9010.71.3.500.

[jan16599-bib-0008] Fritz, M. S. , and D. P. MacKinnon . 2007. “Required Sample Size to Detect the Mediated Effect.” Psychological Science 18, no. 3: 233–239. 10.1111/j.1467-9280.2007.01882. 17444920 PMC2843527

[jan16599-bib-0009] Gabelaia, I. , and R. Bagociunaite . 2023. “The Impact of ‘Quiet Quitting’ on Overall Organizational Behavior and Culture.” Paper presented at the International Conference on Reliability and Statistics in Transportation and Communication.

[jan16599-bib-0010] Galanis, P. , A. Katsiroumpa , I. Vraka , et al. 2023. “The Influence of Job Burnout on Quiet Quitting Among Nurses: The Mediating Effect of Job Satisfaction.” Preprint. 10.21203/rs.3.rs-3128881/v1. 40937525

[jan16599-bib-0011] Galanis, P. , I. Moisoglou , A. Katsiroumpa , et al. 2024. “Impact of Workplace Bullying on Quiet Quitting in Nurses: The Mediating Effect of Coping Strategies.” Healthcare 12, no. 7: 797. 10.3390/healthcare12070797. 38610219 PMC11011316

[jan16599-bib-0012] Galanis, P. , I. Moisoglou , M. Malliarou , et al. 2024. “Quiet Quitting Among Nurses Increases Their Turnover Intention: Evidence From Greece in the Post‐COVID‐19 Era.” Healthcare 12, no. 1: 79. 10.3390/healthcare12010079. PMC10779139 38200985

[jan16599-bib-0013] Galanis, P. , I. Moisoglou , I. V. Papathanasiou , et al. 2024. “Association Between Organizational Support and Turnover Intention in Nurses: A Systematic Review and Meta‐Analysis.” Healthcare (Basel) 12, no. 3: 291. 10.3390/healthcare12030291. 38338176 PMC10855592

[jan16599-bib-0014] George, D. , and P. Mallery . 2010. SPSS for Windows Step by Step: A Simple Guide and Reference, 17.0 Update. 10a ed. Boston: Pearson.

[jan16599-bib-0015] Gorgulu, O. , and A. Akilli . 2017. “The Determination of the Levels of Burnout Syndrome, Organizational Commitment, and Job Satisfaction of the Health Workers.” Nigerian Journal of Clinical Practice 20, no. 1: 48–56. 10.4103/1119-3077.180051. 27958246

[jan16599-bib-0016] Gupta, V. , U. A. Agarwal , and N. Khatri . 2016. “The Relationships Between Perceived Organizational Support, Affective Commitment, Psychological Contract Breach, Organizational Citizenship Behaviour and Work Engagement.” Journal of Advanced Nursing 72, no. 11: 2806–2817. 10.1111/jan.13043. 27293180

[jan16599-bib-0017] Hamouche, S. , C. Koritos , and A. Papastathopoulos . 2023. “Quiet Quitting: Relationship With Other Concepts and Implications for Tourism and Hospitality.” International Journal of Contemporary Hospitality Management 35: 4297–4312. 10.1108/IJCHM-11-2022-1362.

[jan16599-bib-0018] Hiltunen, H. 2023. “Quiet Quitting Phenomenon in Finnish Aviation Industry.” Bachelor thesis, Haaga‐Helia University of Applied Sciences Degree in Aviation Business.

[jan16599-bib-0019] Hu, L. t. , and P. M. Bentler . 1999. “Cutoff Criteria for Fit Indexes in Covariance Structure Analysis: Conventional Criteria Versus New Alternatives.” Structural Equation Modeling: A Multidisciplinary Journal 6, no. 1: 1–55.

[jan16599-bib-0020] Jun, J. , M. M. Ojemeni , R. Kalamani , J. Tong , and M. L. Crecelius . 2021. “Relationship Between Nurse Burnout, Patient and Organizational Outcomes: Systematic Review.” International Journal of Nursing Studies 119: 103933. 10.1016/j.ijnurstu.2021.103933. 33901940

[jan16599-bib-0021] Kang, J. , H. Kim , and O.‐H. Cho . 2023. “Quiet Quitting Among Healthcare Professionals in Hospital Environments: A Concept Analysis and Scoping Review Protocol.” BMJ Open 13, no. 11: e077811. 10.1136/bmjopen-2023-077811. PMC10660974 37984954

[jan16599-bib-0022] Kwak, C. , B. Y. Chung , Y. Xu , and C. Eun‐Jung . 2010. “Relationship of Job Satisfaction With Perceived Organizational Support and Quality of Care Among South Korean Nurses: A Questionnaire Survey.” International Journal of Nursing Studies 47, no. 10: 1292–1298. 10.1016/j.ijnurstu.2010.02.014. 20303081

[jan16599-bib-0023] Li, X. , Y. Zhang , D. Yan , F. Wen , and Y. Zhang . 2020. “Nurses' Intention to Stay: The Impact of Perceived Organizational Support, Job Control and Job Satisfaction.” Journal of Advanced Nursing 76, no. 5: 1141–1150. 10.1111/jan.14305. 31957044

[jan16599-bib-0024] Liu, W. , S. Zhao , L. Shi , et al. 2018. “Workplace Violence, Job Satisfaction, Burnout, Perceived Organisational Support and Their Effects on Turnover Intention Among Chinese Nurses in Tertiary Hospitals: A Cross‐Sectional Study.” BMJ Open 8, no. 6: e019525. 10.1136/bmjopen-2017-019525. PMC6009508 29886440

[jan16599-bib-0025] Liu‐Lastres, B. , O. M. Karatepe , and F. Okumus . 2024. “Combating Quiet Quitting: Implications for Future Research and Practices for Talent Management.” International Journal of Contemporary Hospitality Management 36, no. 1: 13–24. 10.1108/IJCHM-08-2023-1317.

[jan16599-bib-0026] Lowe, M. A. , A. Prapanjaroensin , M. A. Bakitas , et al. 2020. “An Exploratory Study of the Influence of Perceived Organizational Support, Coworker Social Support, the Nursing Practice Environment, and Nurse Demographics on Burnout in Palliative Care Nurses.” Journal of Hospice & Palliative Nursing 22, no. 6: 465–472. 10.1097/NJH.0000000000000686. 32976315

[jan16599-bib-0027] Mahand, T. , and C. Caldwell . 2023. “Quiet Quitting—Causes and Opportunities.” Business and Management Researches 12, no. 1: 9–18.

[jan16599-bib-0028] Moon, Y.‐K. , K. E. O'Brien , and K. J. Mann . 2023. “The Role of Extraversion in the Great Resignation: A Burnout‐Quitting Process During the Pandemic.” Personality and Individual Differences 205: 112074. 10.1016/j.paid.2022.112074.

[jan16599-bib-0029] Page, K. , and N. Graves . 2021. “A Cross Sectional Study of Organizational Factors and Their Impact on Job Satisfaction and Emotional Burnout in a Group of Australian Nurses: Infection Control Practitioners.” BMC Health Services Research 21, no. 1: 441. 10.1186/s12913-021-06477-2. 33971860 PMC8108460

[jan16599-bib-0030] Patrick, A. , and H. K. S. Laschinger . 2006. “The Effect of Structural Empowerment and Perceived Organizational Support on Middle Level Nurse Managers' Role Satisfaction.” Journal of Nursing Management 14, no. 1: 13–22. 16359442 10.1111/j.1365-2934.2005.00600.x

[jan16599-bib-0031] Pevec, N. 2023. “The Concept of Identifying Factors of Quiet Quitting in Organizations: An Integrative Literature Review.” Challenges of the Future 2: 128–147.

[jan16599-bib-0046] Pines, A. M. 2005. “The Burnout Measure Short Version (BMS).” International Journal of Stress Management 12, no. 1: 78–88. 10.3390/ijerph15020344.

[jan16599-bib-0047] Pines, A. M. , and E. Aronson . 1988. Career Burnout: Causes and Cures. New York: Free Press.

[jan16599-bib-0032] Preacher, K. J. , and A. F. Hayes . 2008. “Asymptotic and Resampling Strategies for Assessing and Comparing Indirect Effects in Multiple Mediator Models.” Behavior Research Methods 40, no. 3: 879–891. 10.3758/brm.40.3.879. 18697684

[jan16599-bib-0033] Salehi, T. , M. Barzegar , M. Saeed Yekaninejad , and H. Ranjbar . 2020. “Relationship Between Healthy Work Environment, Job Satisfaction and Anticipated Turnover Among Nurses in Intensive Care Unit (ICUs).” Annals of Medical and Health Sciences Research 10, no. 2: 825–826.

[jan16599-bib-0034] Sasso, L. , A. Bagnasco , G. Catania , et al. 2019. “Push and Pull Factors of Nurses' Intention to Leave.” Journal of Nursing Management 27, no. 5: 946–954. 10.1111/jonm.12745. 30614593

[jan16599-bib-0035] Scheyett, A. 2023. Quiet Quitting. Vol. 68, 5–7. Oxford: Oxford University Press.

[jan16599-bib-0036] Soto‐Rubio, A. , M. D. C. Giménez‐Espert , and V. Prado‐Gascó . 2020. “Effect of Emotional Intelligence and Psychosocial Risks on Burnout, Job Satisfaction, and Nurses' Health During the Covid‐19 Pandemic.” International Journal of Environmental Research and Public Health 17, no. 21: 7998. 10.3390/ijerph17217998. 33143172 PMC7663663

[jan16599-bib-0037] Sousa, K. H. J. F. , R. C. G. Zeitoune , L. F. Portela , G. M. P. Tracera , K. G. Moraes , and R. F. S. Figueiró . 2020. “Factors Related to the Risk of Illness of Nursing Staff at Work in a Psychiatric Institution.” Revista Latino‐Americana de Enfermagem 28: e3235. 10.1590/1518-8345.3454.3235. 32022152 PMC7000181

[jan16599-bib-0038] Tabachnick, B. G. , L. S. Fidell , and J. B. Ullman . 2013. Using Multivariate Statistics. Vol. 6. Boston, MA: Pearson.

[jan16599-bib-0039] Tang, Y. , Y. Wang , H. Zhou , J. Wang , R. Zhang , and Q. Lu . 2023. “The Relationship Between Psychiatric Nurses' Perceived Organizational Support and Job Burnout: Mediating Role of Psychological Capital.” Frontiers in Psychology 14: 1099687. 10.3389/fpsyg.2023.1099687. 36895741 PMC9989200

[jan16599-bib-0040] Tsemach, S. , and A. Barth . 2023. “Authentic Leadership as a Predictor of Organizational Citizenship Behaviour and Teachers' Burnout: What's ‘Quiet quitting' Got to Do With It?” Educational Management Administration & Leadership. Published ahead of print. 10.1177/17411432231212288.

[jan16599-bib-0041] Türe, A. , and A. Yıldırım . 2018. “The Validity and Reliability of Scale of Perceived Organizational Support for Nursing.” Journal of Health and Nursing Management 5, no. 1: 9–18. 10.5222/SHYD.2018.009.

[jan16599-bib-0042] Watts, J. , N. Robertson , and R. Winter . 2013. “Evaluation of Organisational Culture and Nurse Burnout.” Nursing Management 20, no. 6: 1354–5760. 10.7748/nm2013.10.20.6.24.e1113. 24063341

[jan16599-bib-0043] Xueyun, Z. , A. Al Mamun , M. Masukujjaman , M. K. Rahman , J. Gao , and Q. Yang . 2023. “Modelling the Significance of Organizational Conditions on Quiet Quitting Intention Among Gen Z Workforce in an Emerging Economy.” Scientific Reports 13, no. 1: 15438. 10.1038/s41598-023-42591-3. 37723179 PMC10507021

[jan16599-bib-0044] Yıldırım, M. , Ö. Kaynar , G. Arslan , and F. Chirico . 2023. “Fear of COVID‐19, Resilience, and Future Anxiety: Psychometric Properties of the Turkish Version of the Dark Future Scale.” Journal of Personalized Medicine 13, no. 4: 597. 10.3390/jpm13040597. 37108983 PMC10143929

[jan16599-bib-0045] Zheng, J. , and G. Wu . 2018. “Work‐Family Conflict, Perceived Organizational Support and Professional Commitment: A Mediation Mechanism for Chinese Project Professionals.” International Journal of Environmental Research and Public Health 15, no. 2: 344. 10.3390/ijerph15020344. 29462860 PMC5858413

